# The effectiveness of eHealth self-management interventions in patients with chronic heart failure: Protocol for a systematic review and meta-analysis

**DOI:** 10.1371/journal.pone.0268446

**Published:** 2022-09-29

**Authors:** Siru Liu, Jili Li, Zhan Qu, Runyi Li, Jialin Liu

**Affiliations:** 1 Department of Biomedical Informatics, Vanderbilt University Medical Center, Nashville, Tennessee, United States of America; 2 West China Medical School, Sichuan University, Chengdu, China; 3 Wu Yuzhang Honors’ College, Sichuan University, Chengdu, China; 4 Information Center, West China Hospital, Sichuan University, Chengdu, China; 5 Department of Medical Informatics, West China Medical School, Chengdu, China; Faculdade de Medicina de São José do Rio Preto, BRAZIL

## Abstract

**Purpose:**

The objective of this paper is to design a protocol for a systematic review and meta-analysis on the effectiveness of self-management interventions in patients with chronic heart failure.

**Methods:**

The protocol is developed following the Preferred Reporting Items for Systematic Reviews and Meta-Analyses (PRISMA) guidelines. The protocol has been registered in PROSPERO (CRD42021246973). Base on the population, intervention, comparator, and outcome (PICO) framework, our research questions are: 1) What are the effects of eHealth self-management interventions on patients with chronic heart failure? 2) What factors of interventions might affect outcomes? The process includes: 1) search strategy and inclusion criteria; 2) data extraction; 3) risk of bias assessment and 4) data analysis. Searching process and data extraction will be guided by Cochrane Handbook for Systematic Reviews of Interventions. We will use Cochrane Risk of Bias tool to assess the risk of bias. The data analysis will be performed using Metafor package in R.

**Conclusions:**

This systemic review will synthesize the current evidence and identify gaps. Findings in the meta-analysis will provide guidance for designing a more effective self-management intervention for patients with chronic heart failure in future.

## Introduction

Chronic heart failure (CHF) is a severe long-term disease [1]. Especially in the 21st century, there are more than 26 million patients across the globe, which is an ascending trend year by year [2]. Simultaneously, the main disease is no longer confined to the elderly, while the incidence rate of the younger generation is also greatly escalated. Some experts have already attributed it to a global epidemic [3, 4]. CHF remains a major problem in clinical and public health [5]. It is one of the main causes of hospitalization in the elderly (the number of people hospitalized is increasing every year by more than 1 million [2]). This greatly augments a country’s fiscal expenditure on public health. Only in the United States, 6.2 million American adults aged 20 suffered from CHF during 2013 and 2016 [6], and one in nine people died of heart failure in 2011. Besides, it is anticipated that the prevalence of CHF will continue to increase by 46% to 8.5 million between 2012 and 2030 [7]. As the population ages, the associated cost of government investment is estimated to double from $31 billion in 2012 to $70 billion by 2030 [7].

Self-management intervention is an individuals’ ability to manage personal symptoms conditions, psychological health and lifestyles by utilizing resources [8, 9]. eHealth self-management intervention is the use of information and communications technology (ICT) to support self-management [8, 10]. It includes mobile health technologies or digital technologies for CHF self management (e.g., remote support, virtual reality, online education) [11, 12]. eHealth is used to provide tailored information, reminder, decision support and adapt to patients’ needs through information and communication technology, so as to conduct self-management more effectively [10, 13–16].

Compared with traditional self-management intervention such as medication adherence, exercise training and smoking cessation, eHealth self-management intervention may improve self-management in chronic disease settings more efficiently [17]. eHealth self-management intervention could significantly improve fatigue and self-efficacy of the cancer patients [18] and save cost and be accessible and flexible for patients with somatic diseases [19]. Furthermore, the applications of eHealth such as tele-monitoring and home telehealth have suggested beneficial effects on clinical outcomes of heart failure, including a reduction in mortality, all-cause hospitalization and heart failure hospitalization [20].

Current evidence could suggest that eHealth self-management intervention in patients with heart diseases is effective, but there are some limitations in previous research [21]. First, previous research concentrate on congenital heart disease and not on CHF [22]. Second, several studies focus on the outcomes and approaches of heart failure, neglecting the factors of interventions which might affect outcomes [23]. Third, previous research do not particularly focus on the effects of eHealth [24]. The eHealth self-management interventions have been gradually applied in patients with CHF failure, however, the findings are inconsistent [15, 17]. Furthermore, little is known at this point about the effectiveness and the affecting factors of this approach.

Overall, a systematic review and meta-analysis on eHealth self-management interventions in patients with CHF, including the effectiveness and affecting factors, is lacking. Therefore, we will conduct a systematic review and meta-analysis to identify eHealth self-management interventions for patients with chronic heart failure and assess their effectiveness and potential harm to patients, patient satisfaction, economic costs, and supporting evidence of their validity. This systemic review will identify the gaps and intend to design a more effective method for patients.

## Methods

The systematic review and meta-analysis was registered in PROSPERO network (CRD42021246973). The protocol of systematic review was accomplished according Preferred Reporting Items for Systematic Reviews and Meta-Analyses (PRISMA) guidelines [25]. The whole process includes: 1) search strategy; 2) inclusion and exclusion criteria; 3) data extraction; 4) risk of bias assessment and 5) data analysis. The searching process and data extraction will be guided by Cochrane Handbook for Systematic Reviews of Interventions [26]. We will use the Cochrane Risk of Bias tool to assess the risk of bias. The data analysis will be performed using the Metafor package in R.

### Search strategy

To identify randomized controlled trials that provide self-management interventions to patients with chronic heart failure, we developed the search strategy with a librarian through an iterative process. Search terms consist of three parts using Medical Subject Headings terms and keywords: 1) chronic heart failure; 2) self-management interventions; 3) eHealth and 4) randomized controlled trials. The whole search strings are in S1 File. We will perform a comprehensive search in four databases: PubMed, EMBASE, Cochrane Central Register of Controlled Trials (CENTRAL), and CINAHL. We will contact the original authors if we do not have the access to full-text papers.

### Inclusion and exclusion criteria

Inclusion and Exclusion Criteria are followed the Participants-Intervention-Comparison-Outcome (PICO) framework (see Table 1) [27]. We will only include peer reviewed the paper in English with no publication time restriction. We will exclude protocols, feasibility data, pilot studies, and reviews. Three reviewers (SL, JL, and QZ) will use Rayyan as the platform to perform study screening and selection. After removing duplications, two reviewers will screen the titles and abstract, then screen full-text papers following the inclusion and exclusion criteria independently. The inter-rater reliability will be calculated in the Cohen κ value. The third reviewer will judge the potential disagreements in the screening process. If certain eHealth modalities are found to be unsuitable for meta-analysis in subsequent analyses, after discussion by the research team, these studies will be excluded from the meta-analysis and analyzed using a narrative review.

**Table 1:** Inclusion and exclusion criteria.

Participants (P) are defined as adults (age> = 18 years old) with chronic heart failure. We will exclude studies with children or adolescents.
Intervention (I) includes self-management tools consisting of at least one eHealth component, e.g., internet assisted tools, mobile applications. We will exclude the traditional interventions without using any technology support, e.g., face-to-face meetings.
Comparison (C) is chronic heart failure patients with usual care.
Outcomes (O) consists of process outcomes and patient outcomes. Process outcomes are outcomes related to patients’ behavior, e.g., satisfaction, costs, adherence to medication or therapy. Patient outcomes are measures directly related to the disease, e.g., heart function, readmission, and number of emergency visits.
Design: Only RCTs or cluster RCTs were included

(RCTs: randomized controlled trials)

### Data extraction

For each selected study, we will collect the author(s), publication year, country, study design, number of participants, outcomes, descriptions of the control and the intervention, and the theoretical model. We will calculate Cohen’s d as the effect size. For cluster RCTs, we will use the adjusted value reported in the study. If it is not reported, we will extract the intraclass correlation (ICC) value, mean and standard deviation (for continuous outcomes), or odds ratio (for dichotomous outcomes) to calculate the correct effect size. As a reference, Cohen’s d <0.2 means a small effect, 0.2< = Cohen’s d <0.8 means a medium effect, Cohen’s d >0.8 means a large effect [28].

### Risk of bias assessment

To assess the risk of bias in each study, we will use the Cochrane Collaboration Risk of Bias Tool [29]. This validated tool comprehensively evaluates bias in six directions: 1) randomization, 2) deviations from intended interventions, 3) missing outcomes, 4) outcome measurement, 5) selected reported results, and 6) the time of identification and recruitment of participants (only for cluster RCTs). The study has a high overall bias if it has a high risk in at least one direction. The overall bias is low if the study has low risk in all directions. Otherwise, it has a medium risk of bias.

### Data analysis

Outcomes will be reported in two groups: patient outcomes and process outcomes. We will conduct a meta-analysis to summarize the evidence in previous RCTs. We will apply the random-effects model to control the heterogeneity that existed in different studies. The heterogeneity (*I*^*2*^) will be assessed with the omnibus homogeneity test (Q) with the following metrics: 0%-40% (not important heterogeneity); 30%-60% (moderate heterogeneity); 50%-90% (substantial heterogeneity); and 75%-100% (considerable heterogeneity) [30]. The final summarized effect size will be reported with 95% CI. If it is a positive value, it means the current eHealth self-management has an overall positive impact on outcomes. Subgroup analysis will be performed based on the types of outcomes (process/patient), the method of self-management interventions, and with or without using the theoretical model. To evaluate the publication bias, we will perform the funnel plot and Egger’s test the determine the significance of potential asymmetry. The threshold of a significant P value is 0.05.

### Ethics and dissemination

The IRB approval for the protocol and systematic review is not required. Findings in the systematic literature review and meta-analysis would be submitted to a peer-reviewed journal.

### The status and timeline of the study

The review is ongoing. We expect to complete it and report results in 12 months.

## Discussion

As the widespread application of information technology and artificial intelligence in healthcare, eHealth self-management interventions will play an increasingly important role in disease management. The systematic review and meta-analysis will present strong evidence and insight into how to optimize and further develop the eHealth self-management. It will also improve eHealth self-management practice and evaluate the effects of eHealth self-management intervention. The results will be useful to clinicians, nurses, patients and their families in their understanding of eHealth self-management.

To obtain high-quality evidence, we have formulated it strictly in accordance with the guidelines for systematic review and meta-analysis, which is divided into five processes, so that the whole analysis process is not separated from the original data. Thus, it can provide better guidance for scientific self-management intervention. At the same time, in the search and extraction stage of our data, we and a librarian developed a search strategy through an iterative process, using the selected 80+ keywords to search in four databases (PubMed, EMBASE, CENTAL, and CINAH) (S1 File), and then de-duplicated and screened the search results (with the participation of three reviewers) to ensure the extensiveness and comprehensiveness of the data.

Despite the above mentioning advantages in our research, pivotal challenges still remain in the preparation and implementation of this review protocol and the following meta-analysis. First, there are challenges existing in developing the inclusion and exclusion criteria. Generally speaking, considering the complexity of the pathogenesis of chronic heart failure and the diversity of noun expressions, there exist difficulties screening patients with chronic heart failure. Therefore, careful consideration is taken to ensure the accuracy and representativeness of the evidence to support our research. Second, preliminary search results in the databases revealed more than 1000 clinical trials and research studies, with considerable overlap in the four databases. Therefore, patience and caution are required to ensure the preciseness of the selected research data during the data extraction process. Third, on account of the lack of research on the affecting factors of eHealth self-management interventions in patients with chronic heart failure, the integration and analysis of data is considered to be a challenge. As the literature of clinical trials in this field continues to grow, we will constantly update the retrieval results to ensure the reliability of the results of our meta-analysis.

## Conclusion

This systemic review will synthesize the current evidence and identify gaps. Findings in the meta-analysis will provide guidance for designing a more effective self-management intervention for patients with chronic heart failure in the future.

## Supporting information

S1 Checklist PRISMA-P (Preferred Reporting Items for Systematic review and Meta-Analysis Protocols) 2015 checklist: Recommended items to address in a systematic review protocol*. (DOC)

S1 File Search terms for study screening. (DOCX)
