# Supplementary material for: The effectiveness of eHealth self-management interventions in patients with chronic heart failure: Protocol for a systematic review and meta-analysis
Source: PLoS One. 2022 Sep 29;17(9):e0268446. doi: 10.1371/journal.pone.0268446 (PMC9522291; doi:10.1371/journal.pone.0268446)
Supplement: S1 File — (DOCX) [file pone.0268446.s002.docx]

Supplementary material

Search terms for study screening.

| Databases | Search terms |
| --- | --- |
| PubMed | ((heart failure[MeSH] OR HF[Tiab] OR “heart failure”[tiab] OR “cardiac failure” [tiab] OR “heart decompensation”[tiab])  AND (self-management[MeSH] OR self care[MeSH] OR Patient Education as Topic[MeSH Terms] OR self-manag*[Tiab] OR self-car*[Tiab] OR self-monitor*[Tiab] OR self-administration [Tiab] OR self-medication[Tiab] OR educat*[tiab] OR instruct*[Tiab] OR trained[Tiab] OR “action plan*”[Tiab] OR patient-educat*[Tiab] OR patient-cent*[Tiab] OR councel*[tiab])  AND (Telemedicine[MeSH] Internet[MeSH] OR telemedicine[tiab] OR mobile health[tiab] OR mHealth[tiab] OR telehealth[tiab] OR eHealth [tiab] OR m-health[tiab] OR e-health[tiab] OR home-monitor*[tiab] OR telecardiolog*[tiab] OR teleconsult*[tiab] OR e-consult*[tiab] OR econsult*[tiab] OR telemonitor*[tiab] OR mobile technolog*[tiab] OR internet*[tiab] OR web[tiab] OR SMS[tiab] OR short message service[tiab] OR ambulatory monitor*[tiab] OR outpatient monitor*[tiab] OR remote monitor*[tiab] OR digital health [tiab] OR mobile telephon*[tiab] OR mobile phon*[tiab] OR cell phon* [tiab] OR cellular phon*[tiab] OR cellular telephon*[tiab] OR smartphon*[tiab] OR smart phon*[tiab] OR wearable*[tiab] OR mobile devic*[tiab] OR mobile app*[tiab])  AND (randomized controlled trial[MeSH] OR randomised controlled trial[MeSH] OR controlled clinical trial[MeSH] OR random allocation[MeSH] OR evaluation studies[MeSH] OR intervention studies[MeSH] OR “randomized controlled trial”[Tiab] OR “randomised controlled trial”[Tiab] OR “controlled clinical trial”[Tiab] OR “clinical trial”[Tiab] OR “random allocation”[Tiab] OR intervention[Tiab] OR trial[Tiab] OR trials[Tiab] OR random[Tiab] OR randomized[Tiab] OR randomised[Tiab] OR randomization[Tiab] OR randomisation[Tiab] OR randomizing [Tiab] OR randomising[Tiab] OR randomly[Tiab] OR allocate[Tiab] OR allocated[Tiab] OR allocating[Tiab] OR allocation[Tiab])  NOT (("infant"[mesh] OR "child"[mesh] OR "adolescent"[mesh]) NOT "adult"[mesh])) |
| EMBASE | ((heart failure/de) OR HF:ab,ti OR “heart failure”:ab,ti OR “cardiac failure”:ab,ti OR “heart decompensation”:ab,ti)  AND (self-management/de OR (self care/de) OR (Patient Education as Topic/de) OR self-manag*:ab,ti OR self-car*:ab,ti OR self-monitor*:ab,ti OR self-administration:ab,ti OR self-medication:ab,ti OR educat*:ab,ti OR instruct*:ab,ti OR trained:ab,ti OR “action plan*”:ab,ti OR patient-educat*:ab,ti OR patient-cent*:ab,ti OR councel*:ab,ti)  AND (Telemedicine/de Internet/de OR telemedicine:ab,ti OR (mobile health:ab,ti) OR mHealth:ab,ti OR telehealth:ab,ti OR eHealth:ab,ti OR m-health:ab,ti OR e-health:ab,ti OR home-monitor*:ab,ti OR telecardiolog*:ab,ti OR teleconsult*:ab,ti OR e-consult*:ab,ti OR econsult*:ab,ti OR telemonitor*:ab,ti OR (mobile technolog*:ab,ti) OR internet*:ab,ti OR web:ab,ti OR SMS:ab,ti OR (short message service:ab,ti) OR (ambulatory monitor*:ab,ti) OR (outpatient monitor*:ab,ti) OR (remote monitor*:ab,ti) OR (digital health:ab,ti) OR (mobile telephon*:ab,ti) OR (mobile phon*:ab,ti) OR (cell phon*:ab,ti) OR (cellular phon*:ab,ti) OR (cellular telephon*:ab,ti) OR smartphon*:ab,ti OR (smart phon*:ab,ti) OR wearable*:ab,ti OR (mobile devic*:ab,ti) OR (mobile app*:ab,ti))  AND ((randomized controlled trial/de) OR (randomised controlled trial/de) OR (controlled clinical trial/de) OR (random allocation/de) OR (evaluation studies/de) OR (intervention studies/de) OR “randomized controlled trial”:ab,ti OR “randomised controlled trial”:ab,ti OR “controlled clinical trial”:ab,ti OR “clinical trial”:ab,ti OR “random allocation”:ab,ti OR intervention:ab,ti OR trial:ab,ti OR trials:ab,ti OR random:ab,ti OR randomized:ab,ti OR randomised:ab,ti OR randomization:ab,ti OR randomisation:ab,ti OR randomizing:ab,ti OR randomising:ab,ti OR randomly:ab,ti OR allocate:ab,ti OR allocated:ab,ti OR allocating:ab,ti OR allocation:ab,ti)  NOT (("infant"/de OR "child"/de OR "adolescent"/de) NOT "adult"/de) |
| CENTRAL | ([mh "heart failure"] OR (HF):ti,ab,kw OR (“heart failure”):ti,ab,kw OR (“cardiac failure”):ti,ab,kw OR (“heart decompensation”): ti,ab,kw)  AND ([mh “self-management”] OR [mh “self care”] OR [mh “patient education as topic”] OR (self-manag*):ti,ab,kw OR (self-car*):ti,ab,kw OR (self-monitor*):ti,ab,kw OR (self-administration):ti,ab,kw OR (self-medication):ti,ab,kw OR (educat*):ti,ab,kw OR (instruct*):ti,ab,kw OR (trained):ti,ab,kw OR (“action plan*”):ti,ab,kw OR (patient-educat*):ti,ab,kw OR (patient-cent*):ti,ab,kw OR (councel*):ti,ab,kw) AND ([mh “Telemedicine”] OR [mh “Internet”] OR (telemedicine):ti,ab,kw OR (mobile health):ti,ab,kw OR (mHealth):ti,ab,kw OR (telehealth):ti,ab,kw OR (eHealth):ti,ab,kw OR (m-health):ti,ab,kw OR (e-health):ti,ab,kw OR (home-monitor*):ti,ab,kw OR (telecardiolog*):ti,ab,kw OR (teleconsult*):ti,ab,kw OR (telemonitor*):ti,ab,kw OR (mobile technolog*):ti,ab,kw OR (internet*):ti,ab,kw OR (web):ti,ab,kw OR (SMS):ti,ab,kw OR (short message service):ti,ab,kw OR (ambulatory monitor*):ti,ab,kw OR (outpatient monitor*):ti,ab,kw OR (remote monitor*):ti,ab,kw OR (digital health):ti,ab,kw OR (mobile telephon*):ti,ab,kw OR (mobile phon*):ti,ab,kw OR (cell phon*):ti,ab,kw OR (cellular phon*):ti,ab,kw OR (cellular telephon*):ti,ab,kw OR (smartphon*):ti,ab,kw OR (smart phon*):ti,ab,kw OR (wearable*):ti,ab,kw OR (mobile devic*):ti,ab,kw OR (mobile app*):ti,ab,kw)  AND ([mh “randomized controlled trial”] OR [mh “randomised controlled trial”] OR [mh “controlled clinical trial”] OR [mh “random allocation”] OR [mh “evaluation studies”] OR [mh “intervention studies”] OR (randomized controlled trial):ti,ab,kw OR (randomised controlled trial):ti,ab,kw OR (controlled clinical trial):ti,ab,kw OR (clinical trial):ti,ab,kw OR (random allocation):ti,ab,kw OR (intervention):ti,ab,kw OR (trial):ti,ab,kw OR (trials):ti,ab,kw OR (random):ti,ab,kw OR (randomized): ti,ab,kw OR (randomised):ti,ab,kw OR (randomization):ti,ab,kw OR (randomisation):ti,ab,kw OR (randomizing):ti,ab,kw OR (randomising):ti,ab,kw OR (randomly):ti,ab,kw OR (allocate):ti,ab,kw OR (allocated):ti,ab,kw OR (allocating):ti,ab,kw OR (allocation):ti,ab,kw)  NOT (([mh “infant"] OR [mh “child"] OR [mh "adolescent"]) NOT ([mh "adult"])) |
| CINAHL | ( (MH "Heart Failure") OR TI HF OR AB HF OR TI “heart failure” OR AB “heart failure” OR TI “cardiac failure” OR AB “cardiac failure” OR TI “heart decompensation” OR AB “heart decompensation” )  AND ( (MH "self-management") OR (MH "self care") OR (MH " Patient Education as Topic ") OR TI self-manag* OR AB self-manag* OR TI self-car* OR AB self-car* OR TI self-monitor* OR AB self-monitor* OR TI self-administration OR AB self-administration OR TI self-medication OR AB self-medication OR TI educat* OR AB educat* OR TI instruct* OR AB instruct* OR TI trained OR AB trained OR TI “action plan*” OR AB “action plan*” OR TI patient-educat* OR AB patient-educat* OR TI patient-cent* OR AB patient-cent* OR TI councel* OR AB councel* )  AND ( (MH "Telemedicine") OR (MH "Internet") OR TI telemedicine OR AB telemedicine OR TI mobile health OR AB mobile health OR TI mHealth OR AB mHealth OR TI telehealth OR AB telehealth OR TI eHealth OR AB eHealth OR TI m-health OR AB m-health OR TI e-health OR AB e-health OR TI home-monitor* OR AB home-monitor* OR TI telecardiolog* OR AB telecardiolog* OR TI teleconsult* OR AB teleconsult* OR TI e-consult* OR AB e-consult* OR TI econsult* OR AB econsult* OR TI telemonitor* OR AB telemonitor* OR TI mobile technolog* OR AB mobile technolog* OR TI internet* OR AB internet* OR TI web OR AB web OR TI SMS OR AB SMS OR TI short message service OR AB short message service OR TI ambulatory monitor* OR AB ambulatory monitor* OR TI outpatient monitor* OR AB outpatient monitor* OR TI remote monitor* OR AB remote monitor* OR TI digital health OR AB digital health OR TI mobile telephon* OR AB mobile telephon* OR TI mobile phon* OR AB mobile phon* OR TI cell phon* OR AB cell phon* OR TI cellular phon* OR AB cellular phon* OR TI cellular telephon* OR AB cellular telephon* OR TI smartphon* OR AB smartphon* OR TI smart phon* OR AB smart phon* OR TI wearable* OR AB wearable* OR TI mobile devic* OR AB mobile devic* OR TI mobile app* OR AB mobile app* ) AND ( (MH "randomized controlled trial") OR (MH "randomised controlled trial") OR (MH "controlled clinical trial") OR (MH "random allocation") OR (MH "evaluation studies") OR (MH "intervention studies") OR TI “randomized controlled trial” OR AB “randomized controlled trial” OR TI “randomised controlled trial” OR AB “randomised controlled trial” OR TI “controlled clinical trial” OR AB “controlled clinical trial” OR TI “clinical trial” OR AB “clinical trial” OR TI “random allocation” OR AB “random allocation” OR TI intervention OR AB intervention OR TI trial OR AB trial OR TI trials OR AB trials OR TI random OR AB random OR TI randomized OR AB randomized OR TI randomised OR AB randomised OR TI randomization OR AB randomization OR TI randomisation OR AB randomisation OR TI randomizing OR AB randomizing OR TI randomising OR AB randomising OR TI randomly OR AB randomly OR TI allocate OR AB allocate OR TI allocated OR AB allocated OR TI allocating OR AB allocating OR TI allocation OR AB allocation )  NOT ( ( (MH "infant") OR (MH "child") OR (MH "adolescent") ) NOT (MH "adult ") ) |
